# Supplementary figures and images for: Conditional permeabilization of the P. falciparum plasma membrane in infected cells links cation influx to reduced membrane integrity
Source: PLoS One. 2023 Apr 4;18(4):e0283776. doi: 10.1371/journal.pone.0283776 (PMC10072447; doi:10.1371/journal.pone.0283776)

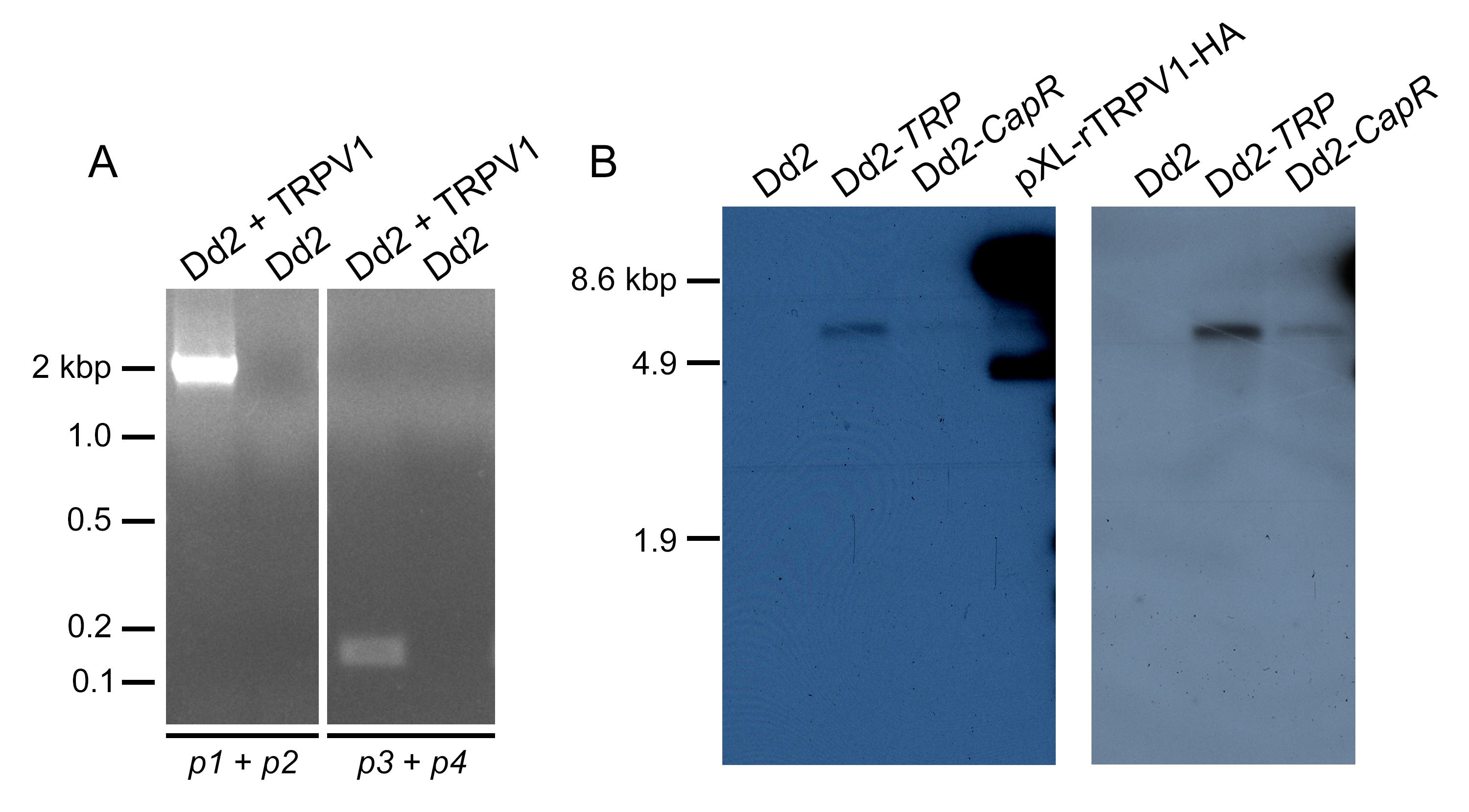

Supplement: S1 Fig — (A) Ethidium-stained gel showing PCR checks for retention of the trpv1 gene in the piggyBac transfectant. Lanes show PCR products using indicated primers for the transfectant line (Dd2 +TRPV1), but not with the untransfected parental control (Dd2). Primer positions are indicated in Fig 1A; sequences are provided in S1 Table. Expected sizes: p1-p2, 1949 bp; p3-p4, 130 bp. (B) Southern blotting showing DNA from indicated parasites or the pXL-rTRPV1-HA transfection plasmid control, each digested with PacI. While the hDHFR probe does not hybridize to DNA from the wild-type Dd2 parent, both Dd2-TRP and Dd2-CapR yield a single band (size ~6 kbp) distinct from the 9.99 kbp seen with the transfection plasmid, indicating a single detected integration into the parasite genome. As the 9.99 kbp band is not detected in Dd2-TRP, this clone does not carry residual episomes. An additional smaller band (< 4.9 kbp) in the plasmid control reflects undigested, supercoiled plasmid. An increased exposure image of the same blot is shown on the right to confirm probe specificity and an unchanged band in Dd2-CapR. (TIF) [file pone.0283776.s002.tif]
