# Supplementary material for: Conditional permeabilization of the P. falciparum plasma membrane in infected cells links cation influx to reduced membrane integrity
Source: PLoS One. 2023 Apr 4;18(4):e0283776. doi: 10.1371/journal.pone.0283776 (PMC10072447; doi:10.1371/journal.pone.0283776)

Fig. 1B blot

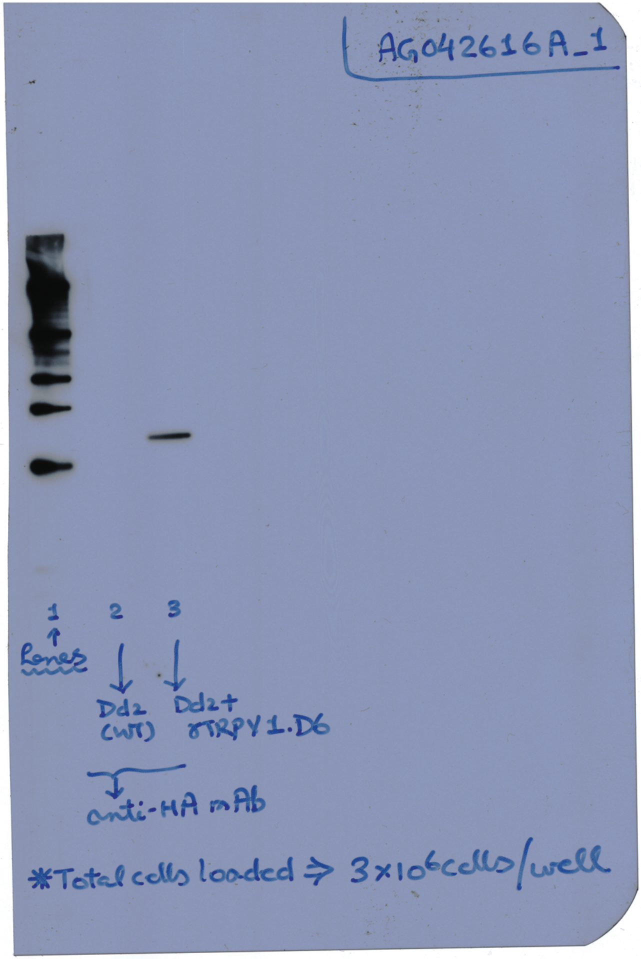
AG042616A_1
1
2
3
lanes
Dd2
(WT)
Dd2+
8TRPY1.D6
anti-HA mAb
*Total cells loaded ⇒ 3×10^6 cells/well

Fig. 1B loading control

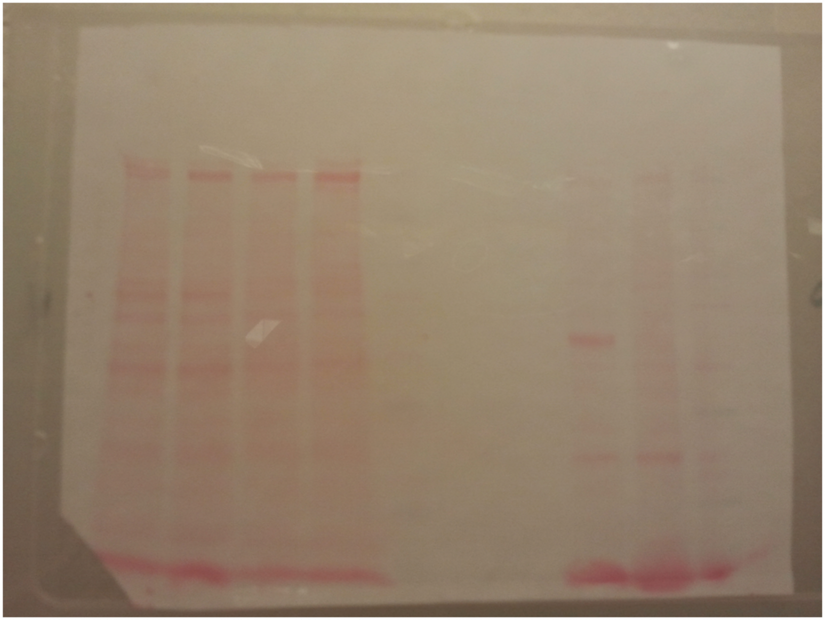

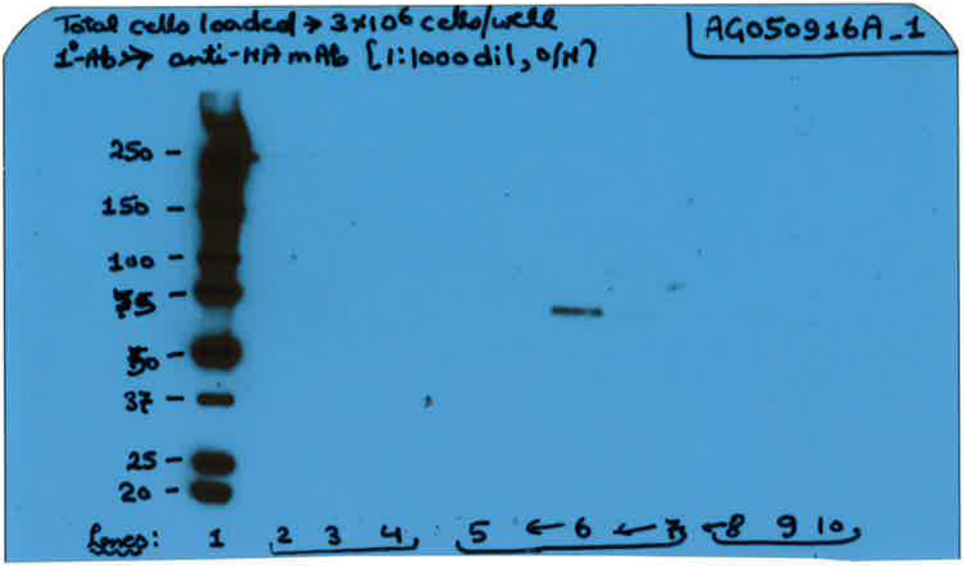
Total cells loaded → 3x10^6 cells/well
1°-Ab → anti-HA mAb [1:1000 dil, O/N]
AG050916A_1
250
150
100
75
50
37
25
20
Lanes: 1 2 3 4 5 6 7 8 9 10

Fig. 1C

Fig. 3

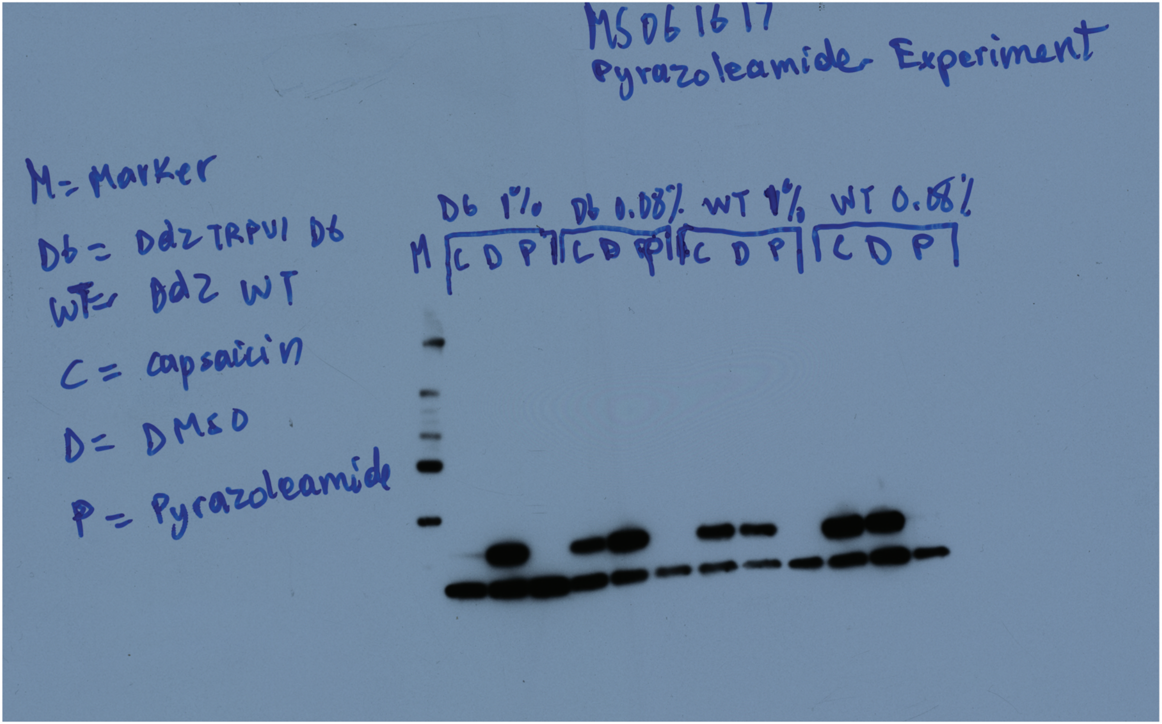
MS061617
Pyrazoleamide Experiment
M = Marker
D6 = Dd2 TRPV1 D6
WT = Dd2 WT
C = capsaicin
D = DMSO
P = Pyrazoleamide
D6 1%
D6 0.08%
WT 1%
WT 0.08%
M
C D P
C D P
C D P
C D P

Fig. 5D

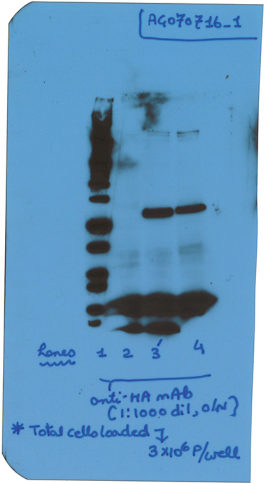
AG070716_1
Lanes 1 2 3 4
anti-HA mAb
(1:1000 dil, O/N)
* Total cells loaded ↓
3×10⁶ P/well

Fig. 5D loading control

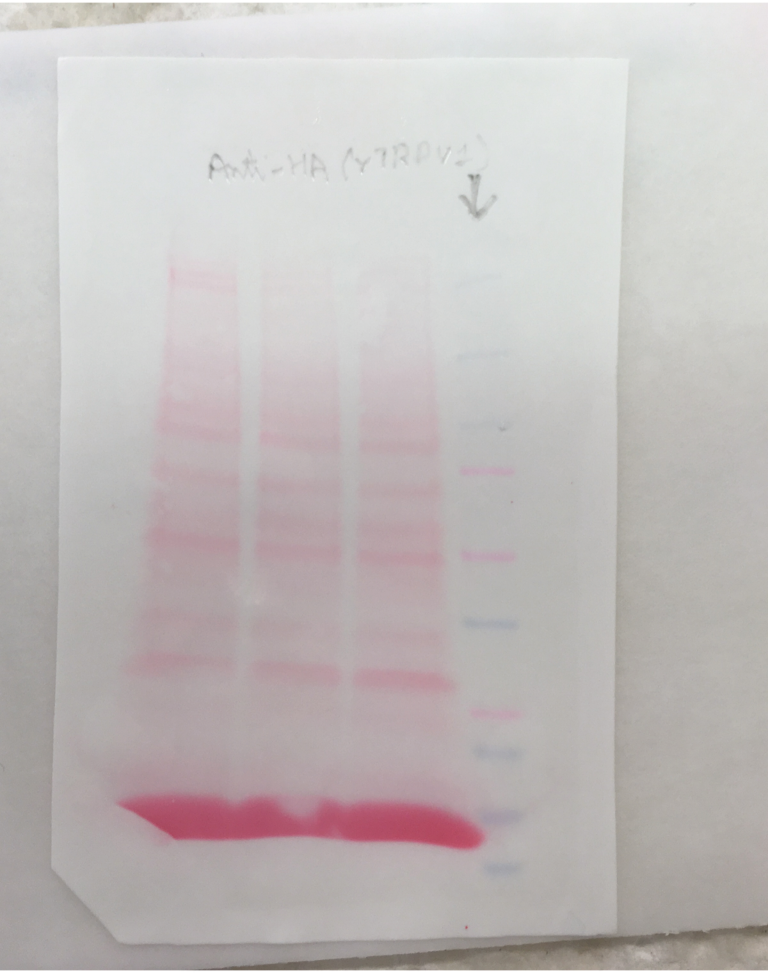

S1A Fig.

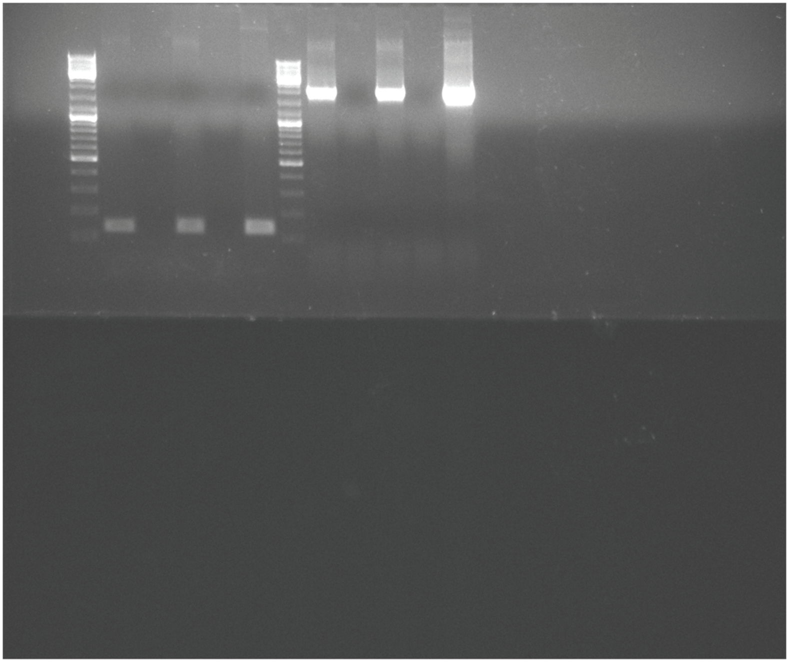

S1B Fig.

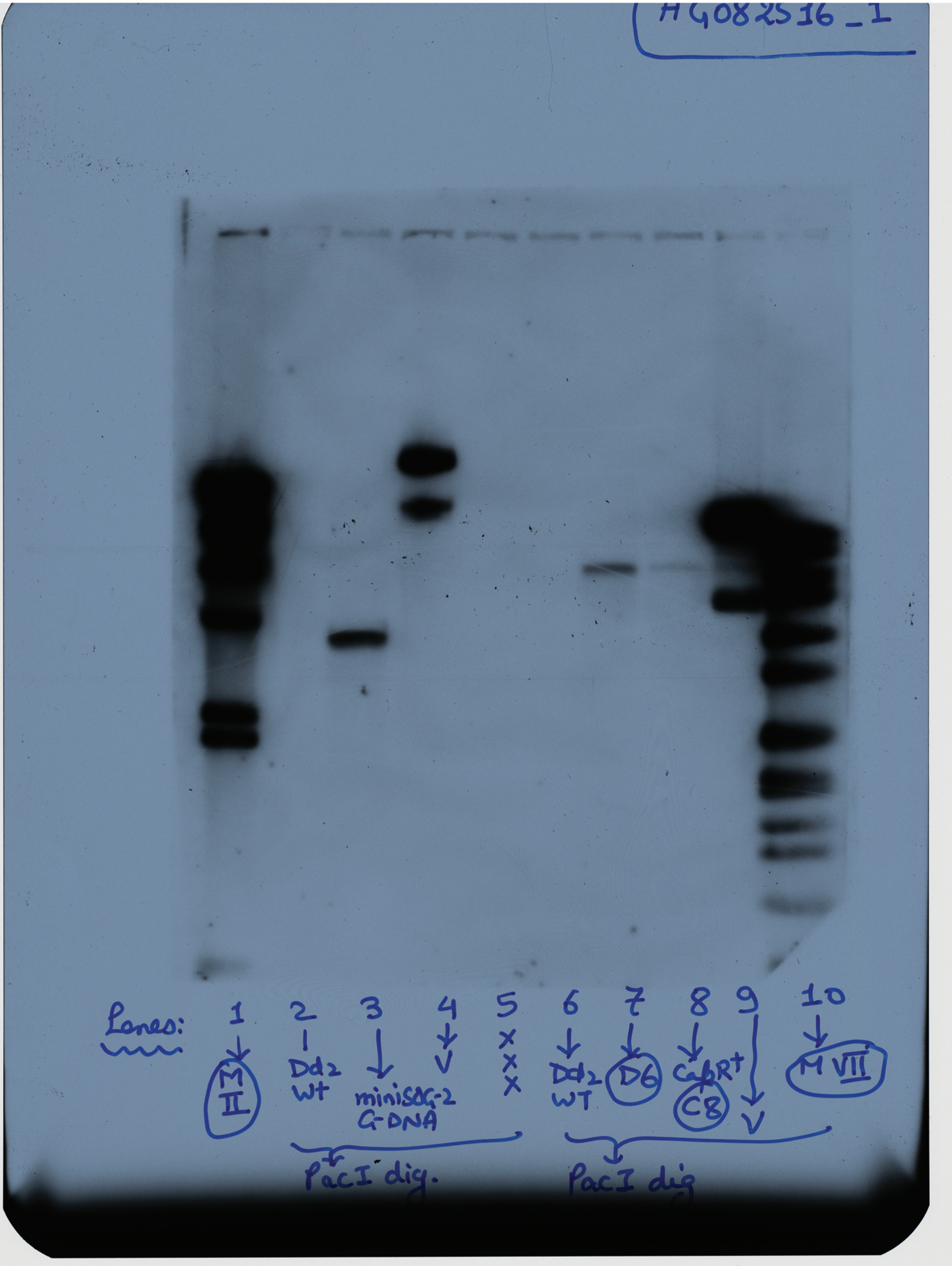
AG082516_1
Lanes: 1 2 3 4 5 6 7 8 9 10
M II
Dd2 WT
miniSOG-2 G-DNA
V
X X X
Dd2 WT
D6
C8
V
M VII
PacI dig.
PacI dig

S1B Fig. (overexposed)

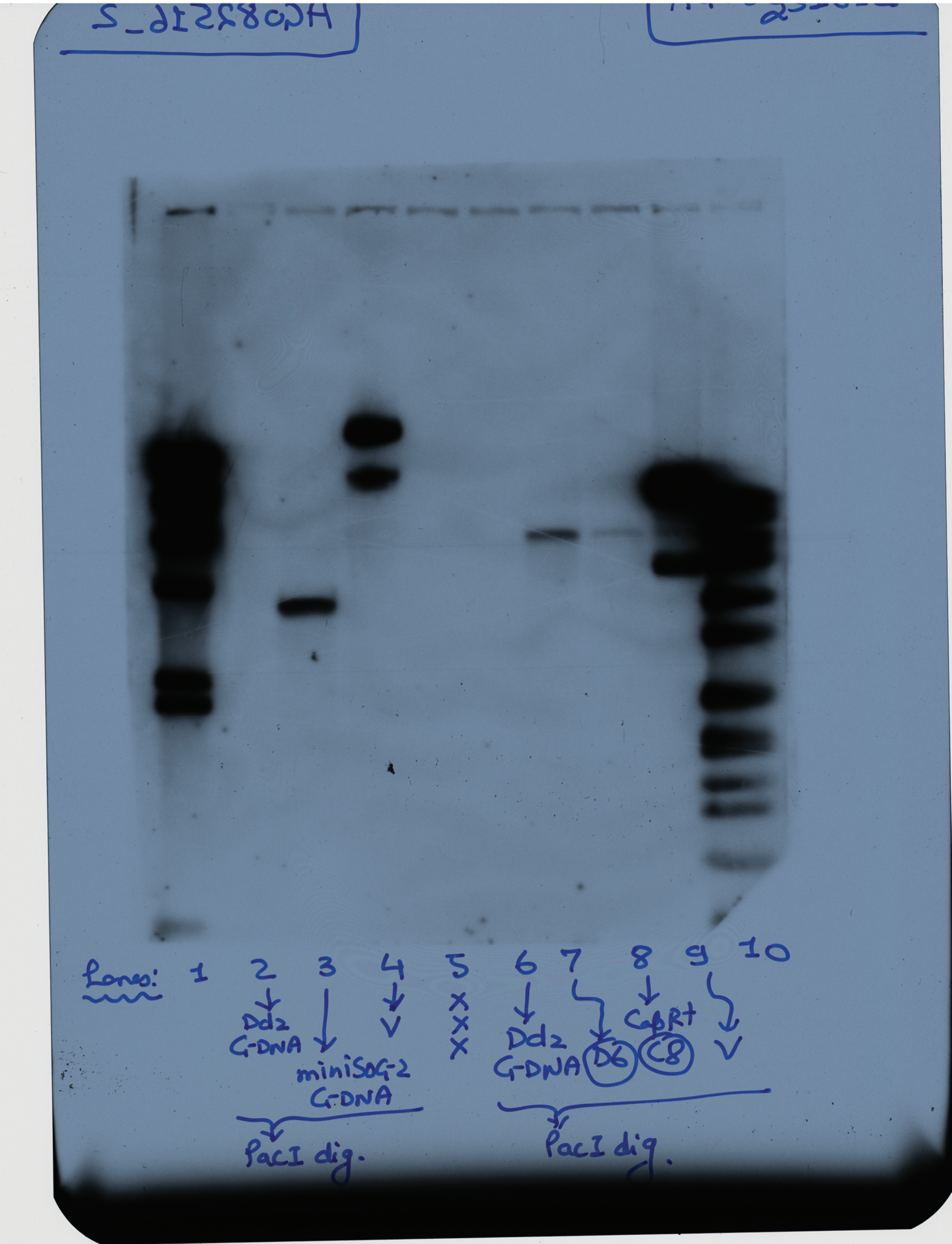

Supplement: S1 Raw images — (PDF) [file pone.0283776.s003.pdf]
