# Supplementary material for: Conditional permeabilization of the P. falciparum plasma membrane in infected cells links cation influx to reduced membrane integrity
Source: PLoS One. 2023 Apr 4;18(4):e0283776. doi: 10.1371/journal.pone.0283776 (PMC10072447; doi:10.1371/journal.pone.0283776)
Supplement: Attachment — Submitted filename: Response to Reviewers.docx [file pone.0283776.s004.docx]

Response to Reviewers

We thank both reviewers for their very positive comments. We have revised the manuscript to address the minor comments raised and hope the paper can now be accepted for publication.

***Reviewer #1:*** *The authors present an elegant an innovative approach to studying the role of malaria ATP4 and the mechanism of action of ATP4 inhibitors. Plasmodium ATP4 has emerged as a key target of pipeline antimalarials, with several distinct classes. The precise mechanism or parasite killing is unclear but ATP inhibitors are known to cause key changes in cellular pH, Na+ levels and plasma membrane composition. This work uses expression of a ligand gated cation channel as a tool to dissect these changes. The resultant transgenic parasites demonstrate selective sensitivity to TRPV1 agonists which result in parasite killing and changes in saponin sensitivity analogous to that seen for ATP4 inhibitors. The work is well written and I have only minor suggestions, mostly surrounding additional discussion of rationale and findings.*

Thank you for your careful read and positive comments.

*Minor comments*

*Technical approach - I am not sure why the authors opted for random integration using the piggyBac system – random integration and potential for variable copy number are big disadvantages, which could be avoided using various Cas9 or even AttB integrase type approaches. These disadvantages are well mitigated by the follow up work, including appropriate genotyping and phenotypic controls. The fact that the integration site is unknown will make these specific lines less attractive for others to use. That said, this does not affect the success of this work in validating the fundamental approach and of course similar lines could be readily remade targeting a known locus. I think the parasite lines will be of broader interest and the methodological development itself provides significant impact, so some discussion of these tool parasites, for example for steps to characterise their integration site/generate separate lines and some additional use case examples would be a good addition.*

We agree with this assessment; our studies were initiated before CRISPR-Cas9 was broadly available and adapted for use in our laboratory. We were using *attB* technology (PMID: 30333047), but had found that the *attP*-carrying plasmid could loop out at low levels due to retention of the Bxb1-expressing episome and, thus, opted for *piggyBac* in this work. Rather than thinking it is a disadvantage, we considered variable copy number of integration with *piggyBac* an advantage because it would allow dose-effect studies if clones with varying numbers of integration events could be obtained, as reported (PMID: 19422698). We have added a new paragraph to the Discussion to include this potential advantage and to propose future studies that capitalize on the advantages of targeted insertion using CRISPR-Cas9 technology. See “ We selected … CRISPR-Cas9 gene insertion.” p. 19.

*Genotyping - The combined genotypic data of PCRs Southerns, and IFA is convincing. If available the Southern blots should include a positive control probe for the DD2 wild type to confirm even DNA loading.*

We ensured uniform loading of genomic DNA with NanoDrop absorbance measurements; this is a standard procedure for quantifying genomic DNA and assessing purity. We have revised the Methods section to clarify that 5 ug of genomic DNA was used for each parasite line. “quantified on a NanoDrop … transfection plasmid”, p. 5. We also revised S1B Fig. to include the Dd2 lane in the overexposed radiograph to further establish that the *hDHFR* probe does not bind to the Dd2 wild type genome; the S1B Fig. legend was modified accordingly “An increased exposure … to confirm probe specificity …”, p. 29.

*Localisation - The EM and IFA images are consistent with membrane localisation, but further IFA images from other parasite stages (particularly rings and late trophozoites) would be informative. It might be easier to discern clear parasite membrane localisation in earlier stages.*

Thank you for this suggestion, which we explored during the course of our studies. The transfection plasmid used the constitutive PF3D7_1132700 promoter to drive *trpv1* gene expression, partly to allow expression throughout the parasite cycle. (We selected this promoter based on unpublished promoter comparison studies performed in our laboratory). IFA using late ring-stage parasites revealed a weak signal that was above background, as shown here (anti-HA recognizing TRPV1 in green, DAPI in blue). A relatively weak signal at early stages is consistent with de novo synthesis in each parasite cycle and increased abundance with parasite maturation due to ongoing transcription and translation under a constitutive promoter.

As shown in this second image, we observed segregation amongst daughter merozoites at the mature schizont parasite stage. We do not think addition of images such as these will add substantively to the story as the EM images provide better evidence of parasite membrane localization. We do not wish to add these images to the manuscript.

*Saponin source - The fact that clear differences in saponin reactivity were seen between different preparation, could the authors comment on that in the discussion. Is there a difference in source/purity etc which could account for this?*

Yes, this is an important point. Commercial saponins are plant-derived mixtures of glycosides, steroidal sapogenins, and other surfactant-like chemicals. Hence, we cannot be sure about why there are batch-dependent effects. Personal communications with the Vaidya lab (who provided significant guidance in establishing the saponin susceptibility assay) suggest that sapogenin content determines efficacy in the aldolase release assay. Admittedly, their assay is phenomenological in nature, but the link to antimalarials targeting PfATP4 and our demonstrated causal relationship to cation leak make it an important observation. We have revised the Discussion to address this key reviewer suggestion. See “Batch-to-batch… advanced antimalarial compounds.” p. 18-19.

*Biochemical characterisation – Although authors discuss the draw backs of some of approaches to measure changes in pH/cation flux in live parasites, it would clearly be an important next step to compare findings with these tool parasites. I don’t think it is necessary to add such experimental data here, but it would be appropriate to lay out next steps for biochemical analysis of the TRPV1 lines in the discussion. All the best, Rob Moon*

We have added “Biochemical studies … homeostasis at the PPM.” to the Discussion. p. 20.

*Reviewer #2: The manuscript “Conditional permeabilization of the P. falciparum plasma membrane in infected cells links cation influx to reduced membrane integrity” by Sylla et al. is a pleasure to read. It describes a very original approach to control sodium permeability of the blood stage malaria parasite plasma membrane by introduction of the TRPV1 channel. This will be a great tool for the research community to explore processes related to the ion homeostasis of the parasite. In the present work the authors used the engineered parasite to test a previously proposed model connecting the function of a PfATP4, a sodium and proton exchange pump, directly with lipid homeostasis. The study is very elegant and every alternative hypothesis that I was able to think of was addressed in the discussion. In my opinion the manuscript can be published as is.*

Thank you for the careful read and these kind words.
